# Supervised Machine Learning Methods and Hyperspectral Imaging Techniques Jointly Applied for Brain Cancer Classification

**DOI:** 10.3390/s21113827

**Published:** 2021-05-31

**Authors:** Gemma Urbanos, Alberto Martín, Guillermo Vázquez, Marta Villanueva, Manuel Villa, Luis Jimenez-Roldan, Miguel Chavarrías, Alfonso Lagares, Eduardo Juárez, César Sanz

**Affiliations:** 1 Research Center on Software Technologies and Multimedia Systems (CITSEM), Campus Sur UPM, Universidad Politécnica de Madrid (UPM), 28031 Madrid, Spain; gemma.urbanos@upm.es (G.U.); a.martinp@upm.es (A.M.); guillermo.vazquez.valle@upm.es (G.V.); marta.villanueva.torres@upm.es (M.V.); manuel.villa.romero@upm.es (M.V.); eduardo.juarez@upm.es (E.J.); cesar.sanz@upm.es (C.S.); 2 Instituto de Investigación Sanitaria Hospital 12 de Octubre (Imas12), 28041 Madrid, Spain; ljimenezr@salud.madrid.org (L.J.-R.); alfonso.lagares@salud.madrid.org (A.L.)

**Keywords:** hyperspectral imaging, machine learning, classification, support vector machine, random forest, convolutional neural network, brain, neurosurgery, tumor

## Abstract

Hyperspectral imaging techniques (HSI) do not require contact with patients and are non-ionizing as well as non-invasive. As a consequence, they have been extensively applied in the medical field. HSI is being combined with machine learning (ML) processes to obtain models to assist in diagnosis. In particular, the combination of these techniques has proven to be a reliable aid in the differentiation of healthy and tumor tissue during brain tumor surgery. ML algorithms such as support vector machine (SVM), random forest (RF) and convolutional neural networks (CNN) are used to make predictions and provide in-vivo visualizations that may assist neurosurgeons in being more precise, hence reducing damages to healthy tissue. In this work, thirteen in-vivo hyperspectral images from twelve different patients with high-grade gliomas (grade III and IV) have been selected to train SVM, RF and CNN classifiers. Five different classes have been defined during the experiments: healthy tissue, tumor, venous blood vessel, arterial blood vessel and dura mater. Overall accuracy (OACC) results vary from 60% to 95% depending on the training conditions. Finally, as far as the contribution of each band to the OACC is concerned, the results obtained in this work are 3.81 times greater than those reported in the literature.

## 1. Introduction

Cancer is caused by the transformation of normal cells into tumor cells. They grow uncontrollably, forming masses called tumors that destroy healthy tissue. Today, cancer is one of the most common causes of death. By 2018, this disease had caused 9.6 million deaths, with more than 18.1 million new cases, and this figure is expected to reach 29.5 million patients per year by 2040 [1]. In the case of brain tumors, glioma is the most common tumor in adults. Unfortunately, glioblastoma multiforme (GBM) or grade 4 glioma (GIV) does not permit long-term survival among patients since it is the most aggressive glioma type [2].

Despite the risks involved, GBM surgery is an unavoidable phase in the treatment of this type of tumor. Surgical intervention in GBM improves survival and patients’ quality of life; nonetheless, it is important to remove as much tumor tissue and as little healthy tissue as possible. The resection is particularly challenging, mainly due to the high infiltration capability of the GBMs, making it particularly difficult to distinguish the boundaries between the tumor and the healthy tissue [3]. Today, new technologies allow the neurosurgeon to perform more complete resections, reducing morbidity. In this type of intervention, the incorporation of neuronavigation tools [4], intra-operative magnetic resonance imaging (iMRI) [5], intraoperative ultrasounds (IOUS) [6] and the use of markers such as 5-aminolevunilic acid (5-ALA) [7] have a decisive influence on the intervention method, allowing the neurosurgeon to perform safer and more effective approaches. Nevertheless, these systems have some disadvantages: firstly, the neuronavigator uses magnetic resonance imaging (MRI) obtained prior to surgery. However, the de-pressurization and cerebrospinal fluid leakage that occur during the craniotomy, combined with the tumor resection and drugs administered [8], may cause brain shifting. Hence, the neuronavigator may not be able to accurately locate the lesion. Secondly, the iMRI solves the problem of brain shifts but increases considerably the operation time, and it also carries the necessity for MRI-compatible surgical equipment [9]. Thirdly, ultrasound images allow the evaluation of this brain displacement during surgery, although they may present artifacts and have a limited resolution [10]. Finally, the use of 5-ALA is an invasive method which could cause side effects to patients and only can be used to detect high-grade tumors [11].

To offer a solution to these problems, in recent years, it has been demonstrated that technologies based on HSI are able to distinguish between in-vivo tumor and healthy tissue during neurosurgery [12]. HSI is an appropriate technology for medical use since it is non-invasive, non-ionizing and does not require any contact with the patient. In addition to neurosurgery, HSI has been used in different types of image-guided surgery to help the surgeon to identify the lesion and its margins, in cases such as abdominal surgeries [13], cholecystectomy [14] and renal surgeries [15,16]. Furthermore, HSI has been applied in other medical applications related to the diagnosis of different kinds of cancer [17,18,19,20,21], retinal diseases [22], diabetes [23] and cardiac diseases [24], among others [25,26]. This is because of the ability of HSI to perceive biochemical and morphological changes associated with the disorder. Furthermore, HSI has been used in other fields such as remote sensing [27], where high dimensional hyperspectral images produce difficulties for land cover classification and dimensionality reduction methods have been proven to help in HSI classification [28]. Additionally, HSI has also been used in food quality analysis [29], among other areas [30,31].

HSI captures the diffuse reflectance per wavelength of the tissue surface in the scene, providing tissue spectral information below and beyond the visual range. The tissues’ spectral signatures are obtained using three-dimensional datasets with spatial and spectral information, called hyperspectral cubes. These cubes are the input for ML algorithms, allowing them to distinguish tissues [32].

ML is a field of computer science—specifically, a subset of artificial intelligence (AI)—that aims to teach computers how to learn through experience in order to predict the actions to be taken without being explicitly programmed. ML algorithms are based on the creation of classification models from data training, which will be analyzed with the aim of predicting new data. ML is subdivided into four types of learning: supervised learning, unsupervised learning, semi-supervised learning and reinforcement learning. Within the supervised learning algorithms, there are two main ML methods: classification and regression. In this work, supervised ML and deep learning (DL) techniques have been applied to train models. The algorithms used for training are support vector machine (SVM), random forest (RF) and convolutional neural networks (CNN).

The decision to use SVM, RF and CNN algorithms is motivated by two main reasons. On one hand, they offer a lower complexity degree compared to other hybrid algorithms [33]. On the other hand, they can obtain results with a high degree of accuracy. It is worth noting that by optimizing the complexity in the implementation of the algorithms, the computational load on the processing systems is reduced. Hence, using the proposed snapshot camera makes it possible to offer results from the entire acquisition chain and present classification in real time. Finally, as can be extracted from the state of the art, these algorithms have proven their reliability when classifying healthy brain tissue and tumor.

First, SVM [34] is a powerful and effective algorithm, being one of the most used predictors applied in classification problems, where the goal is to find the optimal hyperplane separation of the data trained for classifying different classes. Second, RF [35] is a flexible algorithm based on decision trees that operates as an ensemble to achieve a more accurate prediction. Third, CNN [36] is a class of deep learning neural networks (DLNNs) which convolves learned features of the images as input data and uses convolutional layers to classify the different classes effectively. In this way, the classification model is used to predict a finite number of brain tissues using data previously labeled by the neurosurgeon, defined as ground truth (GT) maps. The GT maps contain reference information of the original image taken during surgery to indicate the different types of tissue corresponding to each pixel associated with a spectral signature. This information is used by the ML algorithm and to create in-vivo brain visualizations as classification maps [37,38], providing an accurate tool for the neurosurgeon to excise the tumor. Algorithms used are briefly described in Section 2.5 while comparison results can be found in Section 3.

The contribution of this work is the acquisition of hyperspectral images in an in-vivo surgical environment, their subsequent processing and labeling, as well as the analysis and characterization of spectral signatures. Therefore, three ML algorithms have been adapted to classify tissues of interest in brain tumor neurosurgery. Two evaluation experiments have been designed, the first for intra-patient classification and the second for inter-patient classification. Results from these experiments have been used to compare how the different ML techniques work in order determine the best option for brain tissue classification. The rest of the document is organized as follows: Section 2 describes the materials and methods as well as the data used in this work. Section 3 analyzes the results obtained and provides a brief discussion. Finally, Section 4 clarifies the conclusions and offers future research lines.

## 2. Materials and Methods

This section details the materials and methods used to acquire and process hyperspectral images from the database of brain tissue captures obtained during surgery. In addition, this section describes the classification ML techniques developed, the model selection and the metrics used to evaluate the classifiers’ performance.

### 2.1. Methodology

As an overview, the proposed methodology is based on an acquisition system, described in Section 2.2, from which in-vivo images are captured during neurosurgery. After the raw image acquisition, image pre-processing is carried out, performing HSI cube creation, calibration, spectral correction and image normalization, described in the Section 2.4. Then, outside the operating room, a synthetic RGB image is created and it is used for labeling. The HSI images are labeled by the neurosurgeons, using a labeling tool explained in Section 2.3 for creating GT maps, described also in Section 2.3. These GT maps include 5 class labels: healthy tissue (cerebral gray matter), tumor, venous vessel, arterial vessel and dura mater. From the GT maps, datasets containing the labeled pixel data and pixel labels are created. These datasets are used to train the SVM, RF and CNN algorithms, described in Section 2.5. In order to evaluate the proposed methodology, Experiment A and Experiment B have been carried out:

- Experiment A, described in Section 2.8, consists of using all the images to train, including the image to be classified. In this experiment, 80% of the dataset data is used for training and the other 20% to test the model. For each patient, in experiment A, a 5-fold triple cross-validation, described in Section 2.7, is performed to find the best model. Results from Experiment A could help to determine which algorithm overfits the data, since the classified image has been used for training. As a final result, the metrics explained in Section 2.6 have been used to evaluate the algorithms and the obtained classification maps are presented.
- Experiment B, described in Section 2.8, consists of using all the images for training, except the image to be classified. Thus, in this experiment, 12 images are used to train and 1 to test the model. For each patient, in Experiment B, a 5-fold double cross-validation, described in Section 2.7, is performed to find the best model. This experiment is the one that would actually be performed in real time in the operating room, since a trained model prior to surgery is used to classify a new patient image never seen before by the model. Furthermore, evaluation metrics used to validate the algorithms are explained in Section 2.6 to then present the obtained classification maps.

**Figure 1:**
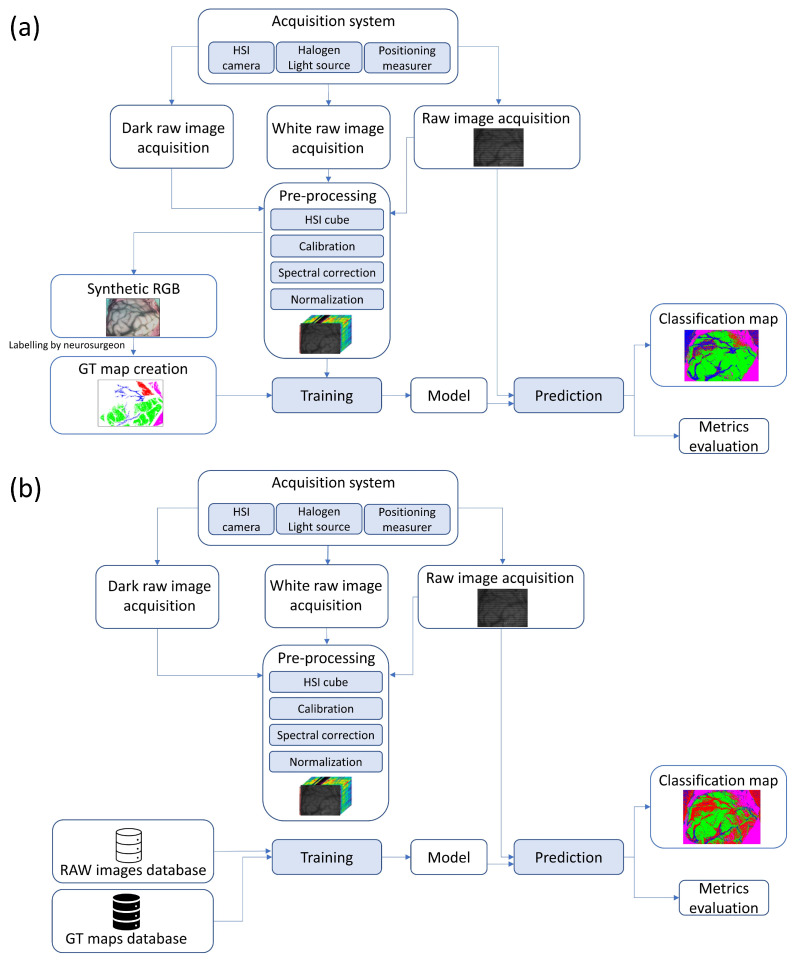
Diagram of the designed methodology for both experiments: (**a**) Experiment A (top), (**b**) Experiment B (bottom).

This methodology is represented in Figure 1, with the experiments A and B being schematized in more detail in Section 2.8. In short, this methodology allows us to:

(i) Evaluate if the classification results obtained with 25 bands from a snapshot HSI camera are comparable to the existing in the state of the art.
(ii) Evaluate the goodness of each of the proposed algorithms and identify under which circumstances they perform better.
(iii) Generate models that can be used to perform real-time classifications of new patients in the operating room.

### 2.2. Hyperspectral Acquisition System

The system used for the data acquisition contains all the elements required to capture hyperspectral images and perform the necessary pre-processing steps, detailed in Section 2.4. It is mainly composed of a hyperspectral camera, a light source, a distance meter, an orientation measuring device and a hyperspectral image acquisition software. In turn, the hyperspectral camera used in this system [39] is based on a snapshot mosaic model with a multi-band matrix sensor, characterized by instantly capturing hyperspectral images without moving the camera. It provides a maximum theoretical ratio of 170 cubes/second scanning capacity, a 217 × 409 pixel spatial resolution and a spectral information distribution in 25 bands along the spectral range from 655 nm to 975 nm. The lens used is a VIS/NIR with 35 mm focal length attached to a cut-off long-pass filter of 650 nm. The camera is characterized by a very small size and weight, 26 × 26 × 26 mm and 32 g, so it is very useful for medical applications. Secondly, the scene illumination is provided by a 150 W halogen light source [40] connected to two fiber-optic cables 180 cm in length and 1.2 cm in diameter, which emits light across the mentioned spectral range where the hyperspectral camera collects information. Thirdly, the camera distance and orientation are not fixed, which makes the procedure for capturing images more flexible, adapting to the conditions of each surgery. The measuring device allows us to know the camera positioning, aiming to pre-process the hyperspectral images properly. In this way, the white reference images were taken at the same distance and angle at which the capture was taken (see Section 2.4). The distance of the hyperspectral captures taken in this work is between 37 cm and 58 cm and the camera orientation is between $25^{\circ }$ and $45^{\circ }$ with respect to the plane where the patient lies. Finally, two software solutions have been used for image acquisition. First, the xiSpec01 software [41] provided by the manufacturer was used. However, later on, a proprietary solution for the acquisition of such images was developed.

The small dimensions of the system facilitate its usage in the surgical reduced environment. The snapshot camera utilized in this work captures HSI cubes in ms. Therefore, results can be shown in real time during surgery. Related state-of-the-art HSI cameras feature a greater number of bands. In [33,42], 128 bands are employed, while the number of bands used in [43] is 826. In contrast, the proposed snapshot camera only captures 25 bands.

### 2.3. Intraoperative Database

Hyperspectral images used for this work were captured from the brain surface during in-vivo neurosurgery procedures, after the completion of the craniotomy and in some cases after the resection of brain tissue, so that the tumor was exposed. All images were taken in the operating theatre using the acquisition system previously described in Section 2.2. Therefore, a database has been created where images with different pathologies have been included. These pathologies are high-grade gliomas (grade III and IV), low-grade gliomas (grade I and II), brain metastases, meningiomas, oligodendrogliomas and aneurysms. More than 50 images of all these pathologies have been accumulated, where 13 hyperspectral images of 12 patients presented in this work correspond to high-grade gliomas with ground truths containing healthy tissue, tumor, venous blood, arterial blood and dura mater labels. These 13 images are the ones used for this study.

The 13 images corresponding to high-grade gliomas (GIII and GBM) used are: ID0018C09, ID0025C02, ID0029C02, ID0030C02, ID0033C02, ID0034C02, ID0035C02, ID0038C02, ID0047C02, ID0047C08, ID0050C02, ID0051C05 and ID0056C02. These patients have been anonymized with a unique reference number in the following format: ID patient number—C capture number. For simplicity, in the rest of the document, these captures will be identified as ID18, ID25, ID29, ID30, ID33, ID34, ID35, ID38, ID47C1, ID47C2, ID50, ID51 and ID56, respectively. With regard to the ID47 patient, there are two existing captures: the first one taken after the duroctomy and the second one taken after a partial brain resection. In both images, the tumor is exposed in different brain levels and therefore they are considered as two cases. All captures have been taken with the same exposure time and aperture, 70 ms and f/4, respectively.

After the image acquisition and the diagnostic confirmation by pathological analysis from ex-vivo tumor samples, the neurosurgeons labeled the different brain tissues from the images. The aim was to create a GT map for each hyperspectral image in order to train supervised ML models. To do so, neurosurgeons have used a tool based on Fabelo et al.’s work [44]. This tool has been further complemented with adaptations for the images captured with the acquisition system described earlier. The tool has been used for sample labeling based on the spectral angle mapper (SAM) classification metric. It calculates the spectral angle between the pixel vector selected and the rest of the end-members, measuring the similarity of each hyperspectral image pixel in relation to the reference pixel selected. In this way, the neurosurgeon manages a threshold, which changes the SAM measure tolerance. In this procedure, 5 reference classes have been defined: healthy brain tissue, tumor, venous blood vessel, arterial blood vessel and dura mater.

**Figure 2:**
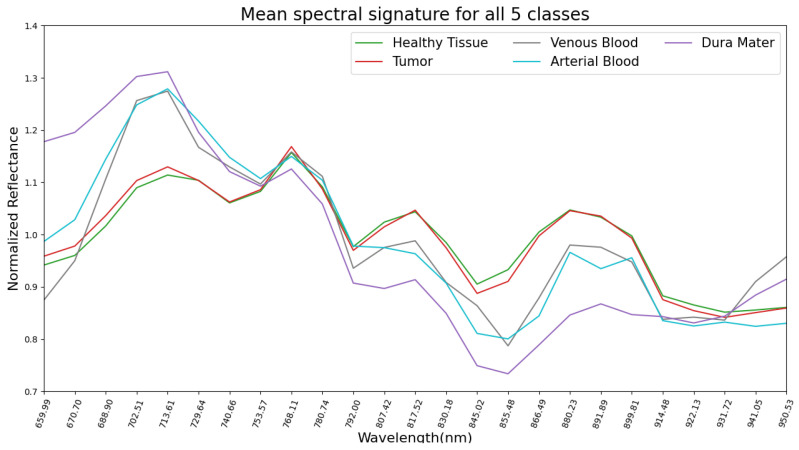
Dataset: representation of mean spectral signatures of all 5 classes.

**Figure 3:**
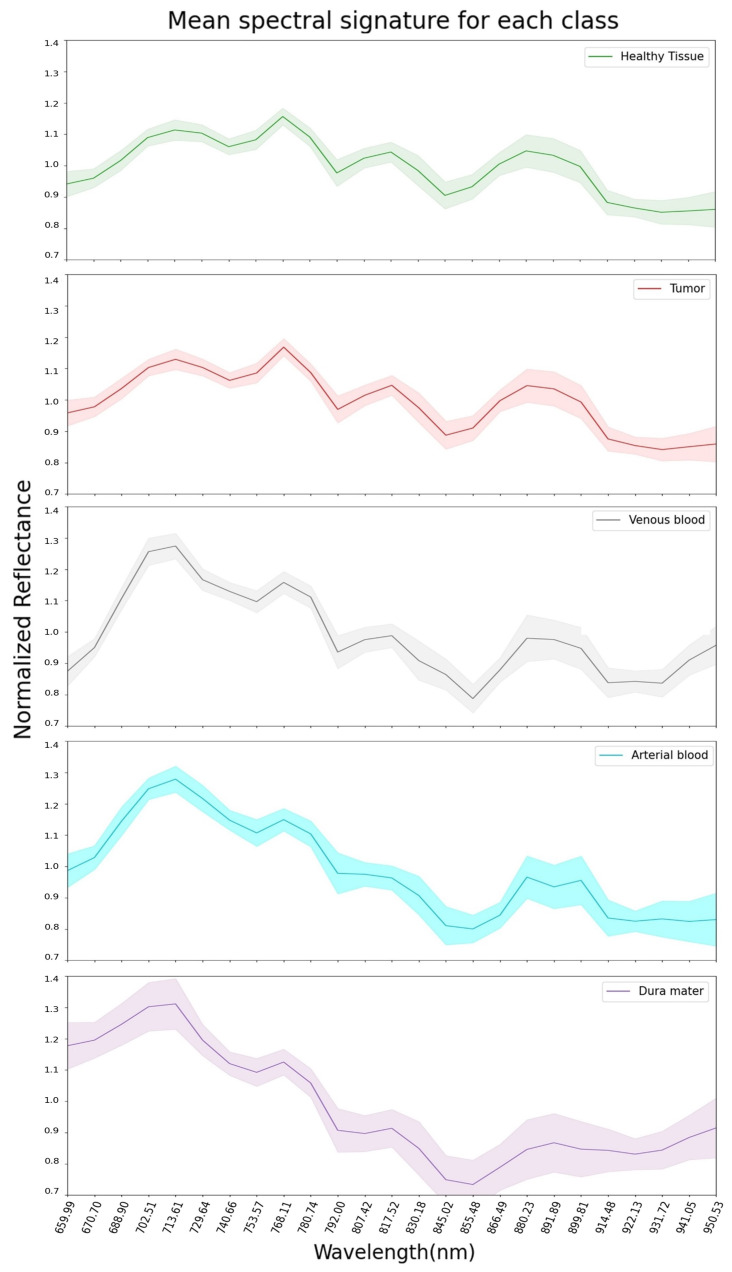
Dataset: representation of mean spectral signatures with standard deviation for each class.

The mean pre-processed (explained in Section 2.4) spectral signatures per pixel of each class from the 13 hyperspectral images used in this study are represented in Figure 2. Here, it can be observed that healthy tissue and tumor classes are very similar across the 660 to 950 nm spectrum. This might explain why SVM, RF and CNN had difficulties in classifying tumors during the experiments for this work, later discussed in Section 3. Regarding the venous blood and arterial blood classes, they both have similar spectral signatures, although they have different tendencies from the healthy tissue and tumor classes. Dura mater’s mean spectral signature is the most distinct one in Figure 2, which might explain the high accuracy prediction over this class with the SVM, RF and CNN trained models. For further analysis, individual mean spectral signatures for each class with their corresponding standard deviations are represented in Figure 3, where both healthy tissue and tumor classes have low standard deviations. Moreover, these two classes have a high number of labeled pixels, as shown in Table 1, with more than 28,000 pixels for healthy tissue and more than 15,000 pixels for tumors. This might indicate that these classes are very consistent across the 660 to 950 nm measured spectrum in this work for all 13 images.

**Figure 4:**
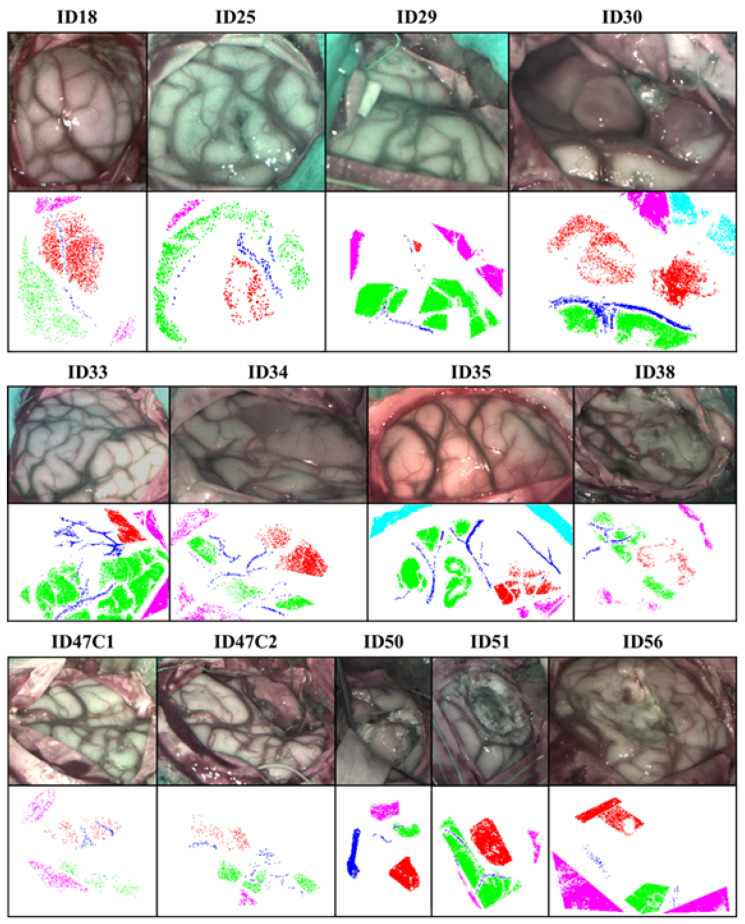
Synthetic RGB and GT maps of patients studied.

Figure 4 shows the synthetic RGB created from a hyperspectral cube band and its corresponding GT map of all patients included in this work. In the GT maps, tumor is represented in red color, venous and arterial blood vessels in deep blue, healthy tissue in green, dura mater in pink color and bone in light blue. The bone class will not be taken into account in this study. All these maps are generated by the neurosurgeon who operated on the patient labeled. Table 1 contains the detailed number of labeled pixels for each class and patient.

### 2.4. Data Pre-Processing

The hyperspectral images have been pre-processed aiming to homogenizing the spectral signatures of the labeled images. The pre-processing chain involves four steps:

1. Hyperspectral cube creation: The raw images obtained with the snapshot camera are converted into hyperspectral cubes. The active area of the hyperspectral raw image has a resolution of 2045 × 1085 pixels and is composed of 25 wavelengths in repetitive mosaic blocks (5 × 5). Therefore, the hyperspectral cube has a spacial resolution of 419 × 217 pixels and a spectral resolution of 25 bands.
2. Calibration: The calibration step is necessary to maintain the reproducibility of the data regardless of the different light conditions of each surgery. To calibrate the images, it is necessary to capture both, white and black reference images. The white reference is obtained using a Lambertian diffuse reflectance target [45], which reflects constantly 95% of the incident light over the entire spectral range covered by the hyperspectral cameras used. The black reference is obtained by covering the camera lens. Regarding white references, a capture is done when the ceramic is placed at the same distance, camera orientation and light conditions as the captured in-vivo brain surface. Applying Equation (1), a band per band calibrated image (Ic) is obtained:
  (1) $$I_{c}=\frac{I-D}{W-D}$$
  where *I* is a band from the captured hyperspectral cube, *D* is the corresponding band from the black reference, and *W* is the same band from the white reference.
3. Spectral correction: The snapshot camera has a high sensibility, so the response curves of the sensor have crosstalk at the peak wavelength of neighbors. In this way, several curves have secondary harmonics that cannot be eliminated with long- or short-pass filters. This effect can be mitigated by the spectral correction process. To obtain the spectral-corrected image, the signal must be multiplied by a correction matrix, as shown in Equation (2):
  (2) $$I_{sc}=I_{c}\times SCM$$
  where $I_{c}$ is a calibrated hyperspectral cube band and SCM is the spectral correction matrix (25 × 25). Each row is a set of virtual band spectral correction coefficients. The correction matrix has been provided by the manufacturer.
4. Data normalization: The normal brain’s curved shape implies that the image pixels are not located at the same height, and therefore the light intensity collected by the camera is different. In this way, when the images are labeled, the tissues will be classified depending on the brightness of each pixel, without taking into account the spectral signatures’ similarity. In order to reduce this effect, it is necessary to use data standardization techniques aiming to preserve the spectral signature shape regardless of the amplitude. The root mean square values (RMS) from spectral signatures over all bands are used as normalizing coefficients (Equation (3)). In Equation (4), these coefficients have been used to normalize the spectral-corrected cube.
  (3) $$coef\left[r,c\right]=\sqrt{\frac{\sum_{b=1}^{B}{\left(I_{sc}\left[r,c,b\right]\right)}^{2}}{B}}$$
  (4) $$I_{Norm}\left[r,c,b\right]=\frac{I_{sc}\left[r,c,b\right]}{coef\left[r,c\right]}$$
  where $I_{sc}$ is the spectral corrected cube with dimensions r×c×b(rows× columns×bands).

### 2.5. Machine Learning Algorithms

In this work, three ML algorithms have been selected: SVM, RF and CNN. GT images, presented in Section 2.3, have been used for training. These images have hundreds or even thousands of pixels labeled by neurosurgeons, meaning that, for those observations, there exists a corresponding labeled output. Algorithms used are briefly described while comparison results can also be found in Section 3.

**Figure 5:**
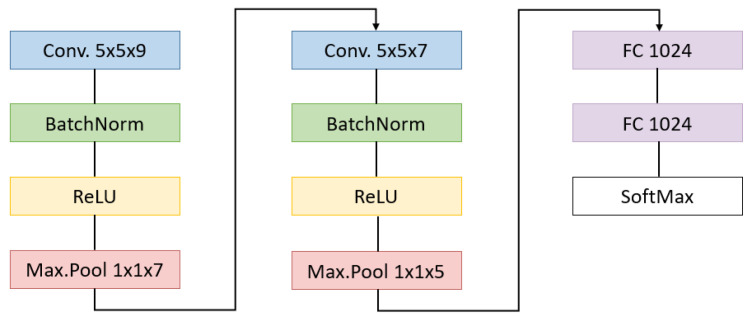
CNN implemented with the filter sizes.

- Support vector machine: SVM is a supervised machine learning algorithm that predicts an optimal hyperplane in an n-dimensional space to divide the training set into multiple classes. Different kernel functions can be specified for the decision function depending on the problem. They could be used to implement a multi-class classification on a dataset, providing in advance a subset of labeled data needed for model training. In previous works related to brain cancer detection [44], different SVM kernels have been used and the best results were obtained with a linear kernel. Therefore, for this work, a linear SVM classifier has been implemented using the Python scikit-learn library [46].
- Random forest: RF is also a supervised machine learning algorithm which uses the statistical resampling technique bootstraping when creating an ensemble of decision trees [35]. During this procedure, the training dataset is sampled with replacement for each tree in the forest and random subsets of features are used at each decision split. Final prediction of the forest depends on which label was classified most by all trees when predicting new data. Published studies about RF have proven it to be a successful classifier when using hyperspectral images [47,48]. RF models have been trained using the aforementioned Python scikit-learn library with 100 trees.
- Convolutional neural networks: CNNs have the characteristic of being composed of two main stages: the feature extraction and the classification processes. In the feature extraction block, the convolutions are performed for detecting patterns in the spatial and spectral dimensions. For a better use of this spectral information, the convolutions can be performed through the spatial coordinates but also along the spectral ones, resulting in a 3D convolutional neural network (3DCNN) like the one used in this paper. This feature extraction stage outputs a vector of reduced characteristics that serves as an input for the classification stage, where a series of fully connected layers map a function to separate the data into the desired classes. Both the parameters of the convolutional and the fully connected layers are trained in a supervised way.
  The network architecture implemented in this work is illustrated in Figure 5. Since deeper architectures showed strong overfitting of the training data, a shallow network structure has been here designed [49]. The number of layers and the network structure are also influenced by the work presented in [33]. The 3DCNN performs pixel-wise classification but, due to its convolutional architecture, it needs the spatial context of the sample to be classified. For this purpose, it receives as input 9 × 9 × 25 overlapping patches from the hyperspectral cube, with the target pixel placed in the center. These dimensions have been empirically set after comparing the accuracy obtained by using patches from 3 × 3 × 25 to 21 × 21 × 25. Its feature extraction stage is composed of two concatenated convolutional blocks. Each one performs a 3D valid convolution followed by a batch normalization layer [50]. The number of filters in the first and second convolution layer is 128 and 256, respectively. A ReLU activation layer has been applied after a max pooling layer that only reduces the spectral information. Then, two fully connected layers converge in the output layer that uses softmax activation for mapping the 5 possible classes. Drop-out values of 0.3% and 0.5% have been chosen for the first fully connected layers.
  The training is performed by gathering the input patches in mini-batches of size 1024, passing two times through the whole set of mini-batches. The AdamW [51] optimizer has been used with a learning rate value of 0.00005 and weight decay of 0.01.

### 2.6. Evaluation Metrics

After training models for each algorithm, a confusion matrix is obtained immediately after predicting either validation or test data. In the confusion matrix, TP stands for true positives, TN for true negatives, FP for false positives and FN for false negatives. Please note that in this work, the main class of interest is tumor. Therefore, TN is any class which was not classified as tumor.

From the confusion matrix, the overall accuracy (OACC) metric is calculated in order to select the best models. This is done during the cross-validation to finally use the elected one to classify new images. The OACC metric is described in Equation (5), where all correct predictions are divided by all predictions made.

(5) $$OACC=\frac{Confusion\;matrix\;main\;diagonal}{Total\;number\;of\;predictions}$$

Three metrics have been used to evaluate the performance of the chosen models after predicting entire patient images. These metrics are accuracy (ACC), sensitivity (SEN) and specificity (SPE), which are described in Equations (6)–(8), respectively:

(6) $$ACC=\frac{TP+TN}{TP+TN+FP+FN}$$

(7) $$SEN=\frac{TP}{TP+FN}$$

(8) $$SPE=\frac{TN}{TN+FP}$$

The ACC metric is useful for determining how well trained models globally classify data. On the other hand, SEN is used to determine how classifiers can correctly predict the class of interest, while SPE indicates the capacity of the models to differentiate properly all classes from the class of interest. Another metric used to evaluate whether models are overfitting is the error metric shown in Equation (9).

(9) $$Error=\frac{FP+FN}{TP+TN+FP+FN}$$

### 2.7. Cross-Validation and Stratified Random Sampling

When utilizing ML algorithms for brain tumor classification, it is necessary to ensure that the trained models do not overfit the training set. Otherwise, these models may lead to poor predictions when classifying new patients’ data. Therefore, cross-validation techniques are used to ensure that the trained models will generalize best when classifying new data. With these techniques, it is necessary to split the data into a training set and a test set before training any model. Once the split is done, the models will be trained with the training set and then they will be evaluated after predicting the test set. There are many ways to train the models: one of them is testing every possible training and test split in the entire dataset, but this may require an exhaustive use of resources. Well-known techniques of exhaustive cross-validation are leave-p-out [52] and leave-one-out [53]. However, a non-exhaustive approach can provide similar results without intensive computing of all the possible ways of splitting the dataset. Two of the most popular non-exhaustive cross-validation techniques are the K-fold method [54] and the Holdout method [55]. For this work, 5-fold cross-validation has been used. Splitting the dataset in equal size of 5 folds results in fewer combinations in which models can be trained and tested. Therefore, for each combination, five folds to train models and test them with the remaining fold have been used.

However, since patient brain images do not have the same amount of pixels for each class, as shown in Table 1, folds may contain huge numbers of pixels from one class while lacking those from others. To solve this issue, the stratified random sampling technique [56] has been applied. By using this sampling method, it is possible to ensure that each fold will approximately have the same amount of pixels for each class.

**Figure 6:**
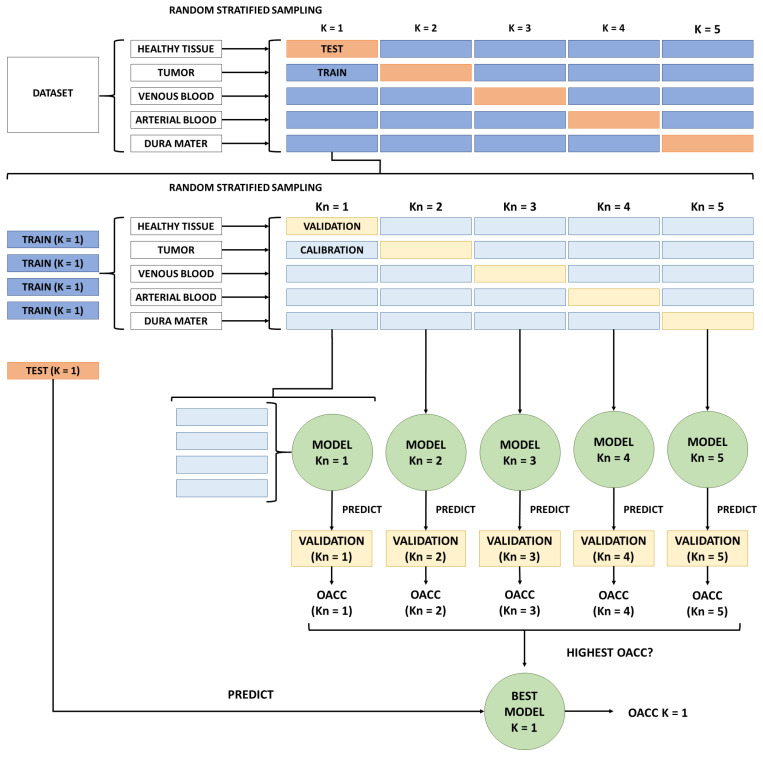
Double cross-validation procedure to train models. For simplicity, the figure only shows external and internal loops for K = 1 combination. K_n_ combinations follow the same procedure even though the figure only shows it for K_n_ = 1.

On the other hand, double and triple 5-fold cross-validations, instead of using a single 5-fold cross-validation, were used for the experiments. Hence, the dataset is split into training and testing sets but into calibration and validation. This method of splitting the dataset into multiple sets removes the introduced bias in variable selection when only using a single training set of fixed composition [57]. This approach, splitting all datasets into folds for double 5-fold cross-validation, is illustrated in Figure 6, describing the external and internal loops used to train the models.

First, desired pixels from patient images are mixed together to then extract all 5 available classes. Once all pixels have been separated by class, they have been randomly split into 5 folds. This way, we ensure that each fold contains 20% of the total pixels for each class. Now, all 5 K combinations would iterate over the external loop to train and test models. However, instead, with the internal loop, all 4 folds composing the training set have been split again in all 5 classes for each K combination. To ensure the usage of stratified random sampling, all pixel classes were randomly selected into 5 sub-folds. It is inside this internal loop where the actual train and test steps happen, using 4 calibration folds and 1 validation fold. Simple maths indicates that 25 models have been trained when double-cross validation has been used. For each K combination, 5 models were trained but only the K_n_ model with the best OACC after predicting the validation fold has been selected. Then, the model with the best OACC predicted its corresponding test fold from its K combination. Please note that the test fold has been isolated until now. This ended up with 5 selected models, which classified their corresponding K test fold, providing 5 different OACC for each K combination. Finally, a comparison between all 5 OACC has been done to select the model with the highest one to use it for the experiments described in Section 2.8.

By adding another external loop before the already described double 5-fold cross-validation, triple 5-fold cross-validation has been implemented. Instead of using the entirety of all patient images as a dataset, this loop has been elaborated to select 80% of every image as dataset for the double cross-validation. For each patient image, all 5 classes have been divided. Then, the stratified random sampling technique has been applied to obtain 5 different folds per patient. The white block entitled Dataset in Figure 6 will be all 4 training folds from all patients to start the double 5-fold cross-validation. Once the double cross-validation is done, the trained model is tested with all testing folds from all patients to obtain an OACC. This strategy is applied for some remaining combinations between all patient images. The criteria of which combinations have been computed consisted of using the same fold grouping for every image. For example, if, for patient image ID18, the first 4 folds have been used for training, only the first 4 folds for every image have been used as the dataset in the double 5-fold cross-validation. Any other fold combination between patients has not been computed, since it would require much more computation power.

### 2.8. Experiments

**Figure 7:**
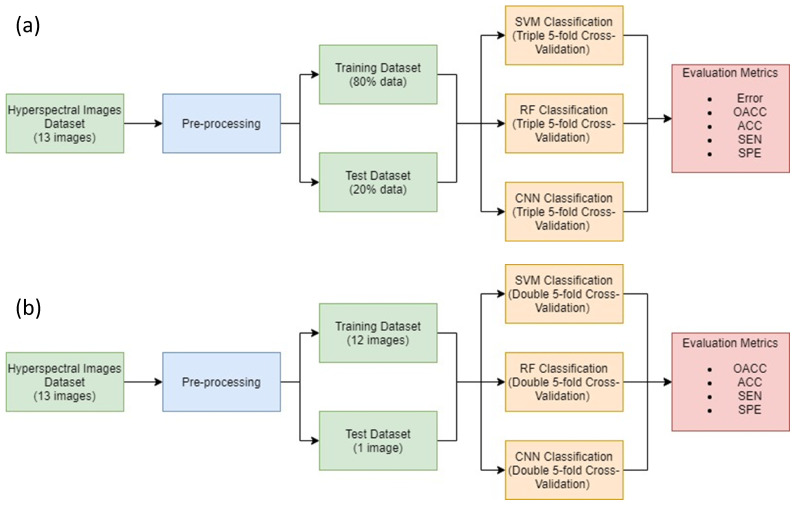
Diagram for both experiments: (**a**) Experiment A (top) and (**b**) Experiment B (bottom).

In order to evaluate the database and the methodology proposed, two experiments have been carried out and are presented in Figure 7. Each experiment has been developed by using all three algorithms previously explained in Section 2.5 (SVM, RF and CNN) with the aim of analyzing the best option for brain cancer detection:

- Experiment A: consists of an intra-patient classification where all patients have been used to train and to test the model. Training dataset consists of 80% of the data while the remaining 20% is used to test the model. The best SVM, RF and CNN models are determined using a triple cross-validation process to elect the best 80% dataset for every patient. In addition, the entirety of each patient image has been labeled with the best models. Since this experiment classifies similar data that have been used for training, the goal is to evaluate which ML algorithm will overfit most to the training set.
- Experiment B: consists of an inter-patient classification. The goal with experiment B is to determine which ML algorithm would generalize best in a real situation, where a trained model is used during surgery to classify a new patient image never seen before. Since thirteen images have been used for these experiments, thirteen models have been trained for every algorithm. For each one, all data from 12 patient images were used for training, while the remaining image was used as a test set. Each of these models has been selected after a double cross-validation to ensure that every pixel has been used as training and test data. Additionally, the remaining image not used during training has been used to classify. This way, we ensure that each image was used for training and testing.

The following section presents the obtained results of both experiments and discussion about them.

## 3. Results and Discussion

First, it is worth remembering that 13 images captured during 12 live surgeries have been used to evaluate both experiments A and B, previously presented in Section 2.3. These images have been used for our experiments since they are the only images which contained GBM pathology as well as healthy tissue, tumor, venous blood, arterial blood and dura mater labeled pixels in their ground truth.

### 3.1. Experiment A

**Figure 8:**
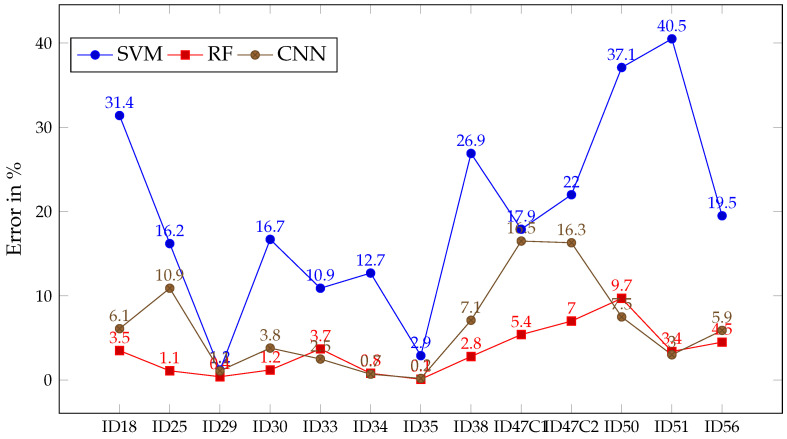
Error in % for SVM (blue), RF (red) and CNN (brown) after classifying each patient image in Experiment A.

As can be seen in Table 2, the OACC metric is presented for every trained ML algorithm after predicting each patient, as well as its mean and standard deviation (SD). The values of OACC obtained per patient provide enough evidence to confirm that the three algorithms are capable of learning from the set of images selected. Therefore, the group of images is suitable, at least, for the training stage in Experiment B. However, the results show some features that may play a role in the subsequent analysis of outcomes of the Experiment B. One of these traits can be observed in Figure 8, concerning the lower error that RF and CNN achieve in almost every single patient compared to SVM. In the case of RF, it is never greater than 10%, whilst its OACC has its lowest at 87.6%, with most of its values above 95%. Regarding CNN, it achieves the highest OACC for three of the patients, keeping an error tendency similar to RF. When it comes to SVM, it shows a different behavior than the other two algorithms. In this case, SVM achieved less than 80% of OACC for most of the patients, alongside an error percentage much higher than RF and CNN. The only exceptions are found for patients ID29 and ID35, where the error and the OACC show similar values for the three algorithms. The flexibility RF and CNN shows that adjusting to the majority of patients entails a possible risk of overfitting that SVM seems less prone to be affected by. Additionally, the disparity in the results obtained by SVM may be interpreted as a sign of the high variability between the characteristics of the patients, which might go unnoticed if only the spectral signatures are observed. This variability will be a strong element in the analysis of Experiment B.

**Figure 9:**
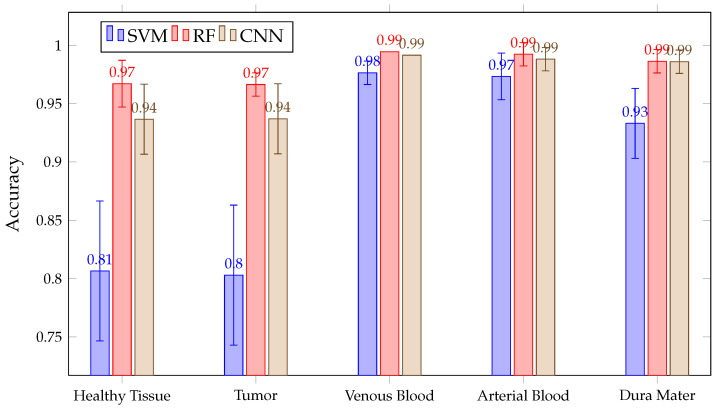
Mean ACC for all patient predictions and every tissue for SVM, RF and CNN algorithms obtained in Experiment A.

**Figure 10:**
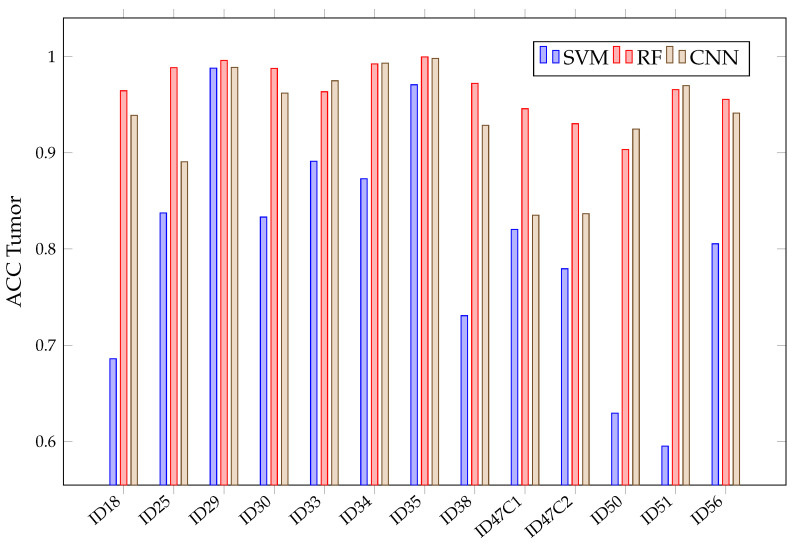
SVM, RF, CNN accuracy in tumor class for each patient in Experiment A.

After acquiring all predictions for all three algorithms, a mean ACC calculation is made for each tissue and all classified patients. This mean ACC is presented in Figure 9. There, it can be seen that RF and CNN have similar ACC results in each tissue after predicting all patients. Meanwhile, SVM achieved a lower ACC than the previous two in the healthy tissue, tumor, venous blood and dura mater classes. Mean ACC Values in RF and CNN are over 90% in all classes, with a fairly stable behavior. This behavior can be observed in Figure 10, where the ACC obtained is presented with the three algorithms per patient in class tumor. However, it can be seen that SVM presents high variability between patients, which explains its high confidence interval in classes such as tumor or healthy tissue.

**Figure 11:**
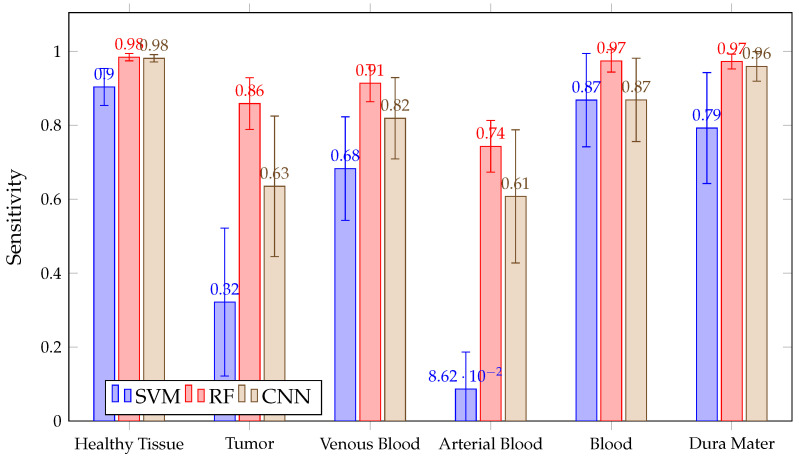
Mean SEN for all patient predictions and every tissue for SVM, RF and CNN algorithms obtained in Experiment A.

**Figure 12:**
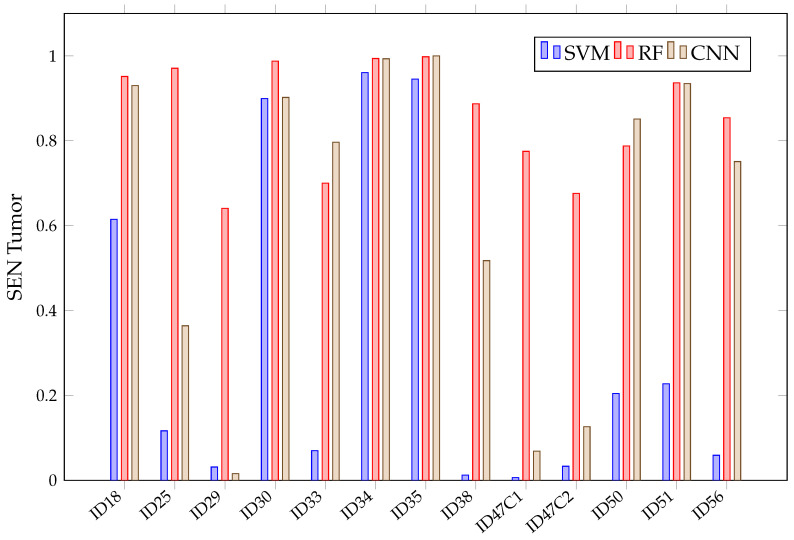
SVM, RF, CNN sensitivity in tumor class for each patient in Experiment A.

When looking at the mean SEN in Figure 11, it can be observed that it is not possible to conclude, among the chosen algorithms, which presents the best sensitivity. This is because of their overlapping confidence intervals in all classes. However, SVM presents worse SEN results in healthy tissue, tumor, arterial, venous blood and dura mater classes than RF. It also has worse SEN than the other algorithms in the healthy and arterial blood classes. Furthermore, SVM again shows a rather unstable behavior; this can be seen in Figure 12, which shows the SEN obtained per patient and algorithm in the tumor class. These results show how SVM obtains results above 80% in patients ID30, ID34 and ID35, but in others, such as ID29, ID33, ID38, ID47C1, ID47C2 and ID56, it shows values below 6%. In addition, CNN presents more variability per patient, especially in the tumor class, venous blood and arterial blood. In Figure 12, it can be observed that CNN obtains SEN results above 80% in patients ID18, ID30, ID34, ID35, ID50 and ID51, but in others, such as ID29, ID47C1 and ID47C2, it does not exceed 13%. However, RF behaves more stably, obtaining sensitivity results above 64% in all patients.

It should be noted that the sensitivity of arterial blood with SVM is very weak, with an average of approximately 8%. This may be due to the fact that the arterial class has very few labeled pixels, as shown in Table 1. This table shows that the maximum percentage of labeled pixels of artery over the total image is 12.46% in patient ID47C2, but in 8 of the 13 patients, it is less than 2%. During the surgery, it is not crucial for the neurosurgeon to differentiate between vein and artery, so further evaluation was performed by merging the arterial and venous blood classes in order to create a single blood class. To obtain the SEN metric of the generic blood class, Equation (10) was used.

(10) $$SENS_{blood}=\frac{TP_{arterial}+TP_{venous}}{\left(TP_{arterial}+FN_{arterial}\right)+\left(TP_{venous}+FN_{venous}\right)}$$

Figure 11 shows how RF achieves a mean SEN greater than 97% for the blood class. The average SEN in SVM and CNN is similar and exceeds 87%, but SVM presents a higher confidence interval than CNN and RF. This indicates that the inter-patient variability of SVM is greater than others in SEN results.

**Figure 13:**
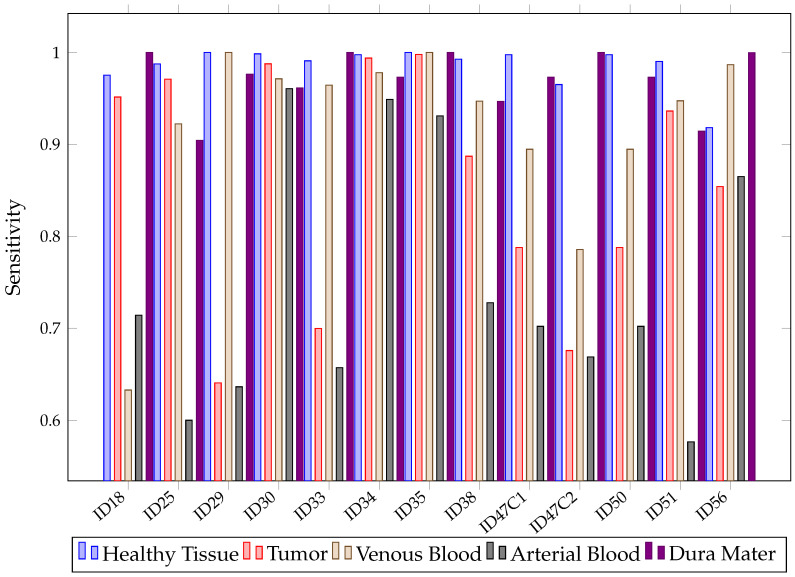
RF sensitivity for each patient and tissue in Experiment A.

In more detail, Figure 13 shows that, for healthy and tumor tissues, RF has achieved SEN values above 74%. Therefore, after evaluating the results for Experiment A, it can be stated that RF tends to overfit. Although models that overfit the training set are not desirable, an opportunity with the RF algorithm can be foreseen due to its high accuracy and sensibility in classifying the five different types of tissues present.

**Figure 14:**
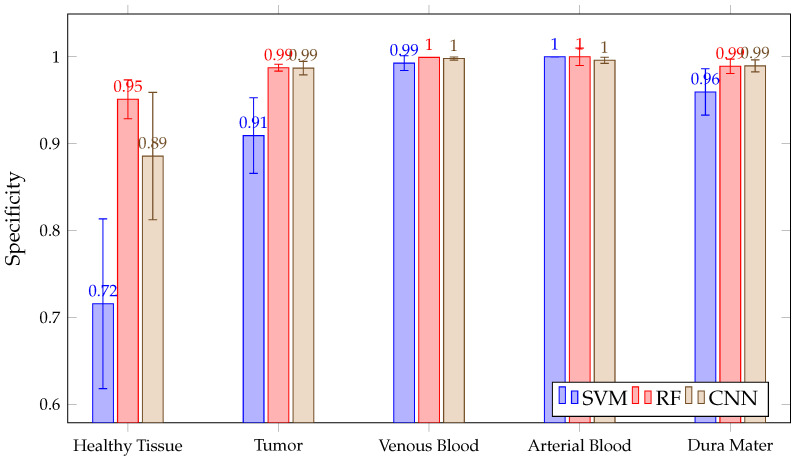
Mean SPEC for all patient predictions and every tissue for SVM, RF and CNN algorithms obtained in Experiment A.

In addition, as shown in Figure 14, all three algorithms have high SPE results, with a mean above 90% in the tumor, venous blood, arterial blood and dura mater classes. However, in the healthy class, SVM presents a lower SPE than RF, with CNN and RF presenting high inter-patient variability in healthy tissue. Moreover, SVM presents lower SPE than RF and CNN in the tumor class.

**Figure 15:**
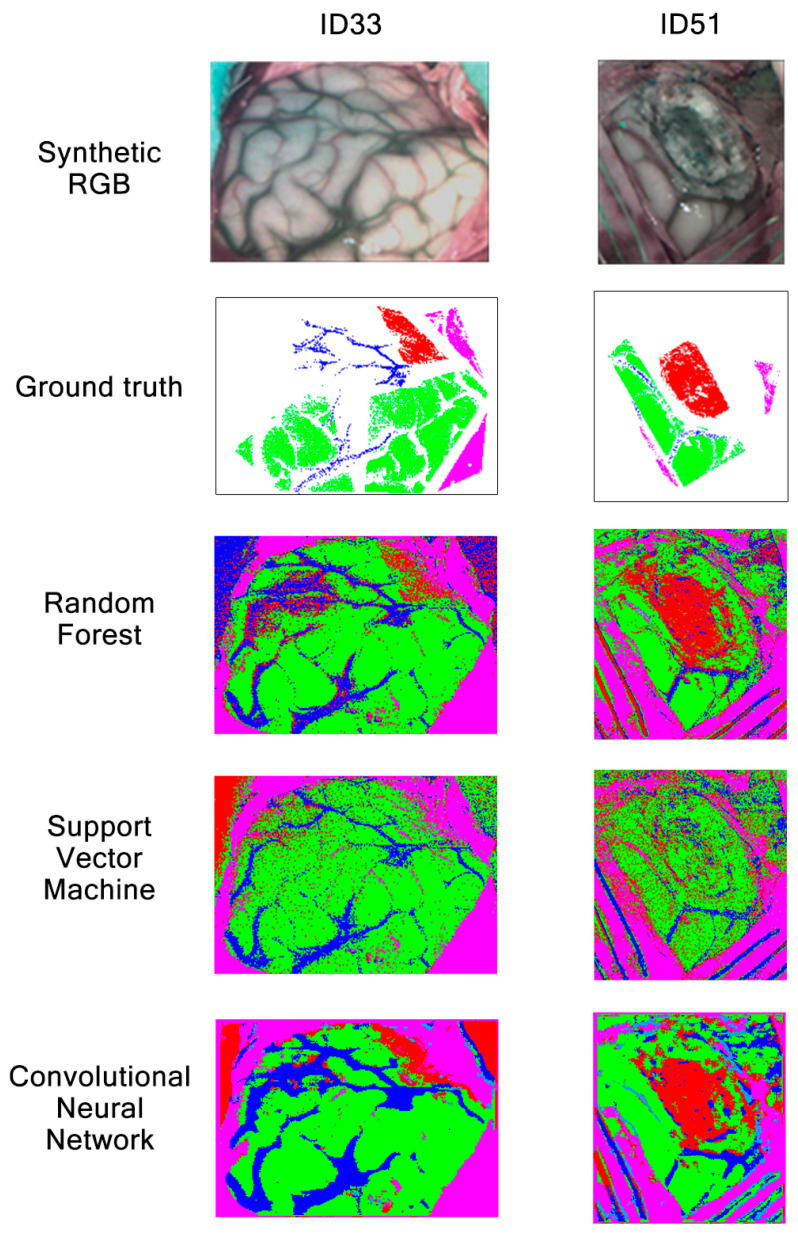
Synthetic RGBs, ground truths and classification maps of patients ID33 and ID51 obtained in Experiment A after being classified by RF, SVM and CNN.

Classification maps obtained with RF, SVM and CNN during Experiment A for patients ID33 and ID51 are shown in Figure 15. Patients’ synthetic RGB and GT maps are also shown as a reference. On one hand, image ID33 contains information regarding the brain surface affected by glioma grade III. On the other hand, image ID51 contains a brain affected by astrocytoma grade III at an advanced stage in the surgical procedure. Regarding label colors, green represents healthy tissue, red indicates tumor, blue indicates venous blood, cyan arterial blood and pink represents dura mater. From these results, we can see how RF, SVM and CNN are able to classify venous blood and dura mater for patient ID33. Although, for this patient, each algorithm has problems differentiating healthy tissue and tumor, CNN provides less incorrectly classified tumor pixels than RF and SVM. This can also be seen in Figure 12, where CNN obtained 10% higher tumor sensitivity than RF and almost 70% higher than SVM for patient ID33. For patient ID51, results show that both RF and CNN can classify tumor tissue located in deeper layers. On the other hand, SVM classifies most tumor as healthy. Again, this can be seen in Figure 12, where SVM obtained around 25% tumor sensitivity while RF and CNN obtained almost 95% for patient ID51. In any case, results show that, for Experiment A, both RF and CNN can provide clearer classification maps than SVM.

### 3.2. Experiment B

**Figure 16:**
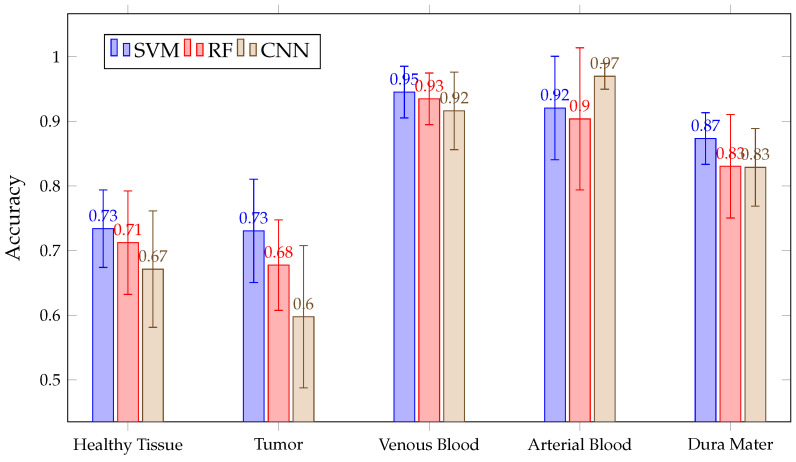
Mean ACC for all patient predictions and every tissue for SVM, RF and CNN algorithms obtained in Experiment B.

**Figure 17:**
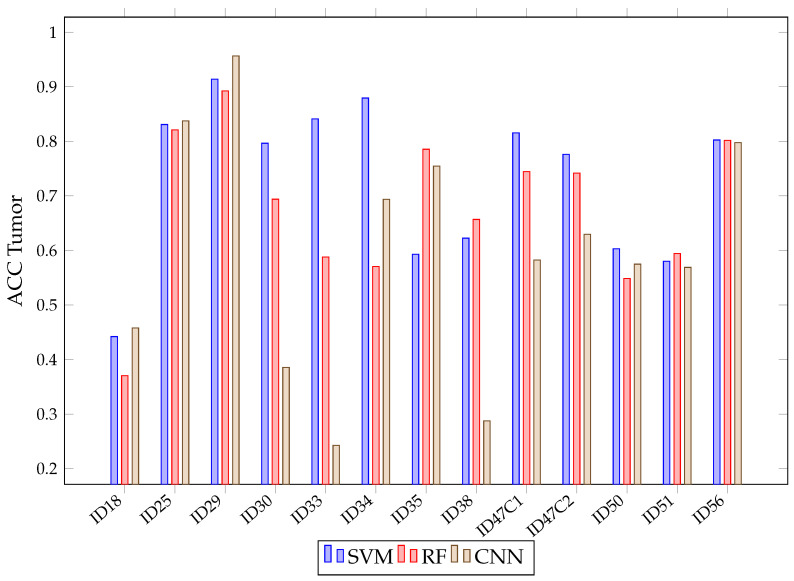
SVM, RF, CNN accuracy in tumor class for each patient in Experiment B.

Figure 16 shows the mean accuracy for each class and algorithm used. All methods (SVM, RF and CNN) present similar results. Although SVM presents better average accuracy results than the rest, the results present overlapping and significantly large confidence intervals because, depending on the patient classified, the accuracy results per class and algorithm vary. This variation is intrinsic to each patient and depends on the patient tissues’ biology. Due to this, we needed to train models with all these images to possibly classify better new patients. Moreover, the confidence intervals are high because the models used are different in the 13 images classified. However, in most cases, results exceeded 60%. The behavior of these algorithms is exemplified in Figure 17, where the results of accuracy per patient in the tumor class are shown. This figure shows that, depending on the patient, one algorithm will classify better than another. For example, it is observed that six patient tumors (ID30, ID33, ID34, ID47C2, ID47C8 and ID50) are better classified with SVM, three of them are better classified with RF (ID35, ID38 and ID51) and two of them obtain better results with CNN (ID18 and ID29). It also shows how the three algorithms obtained very similar ACC results when classifying the tumor class for the remaining patients (ID25 and ID56).

Overall, Table 3 shows how SVM has achieved the best results in 7 out of the 13 predictions, while CNN in 4 and RF in 2, although none of the algorithms achieved OACC values over 80%. Moreover, these results may be related to the high variance in the dataset, which could influence the models’ predictions during training.

**Figure 18:**
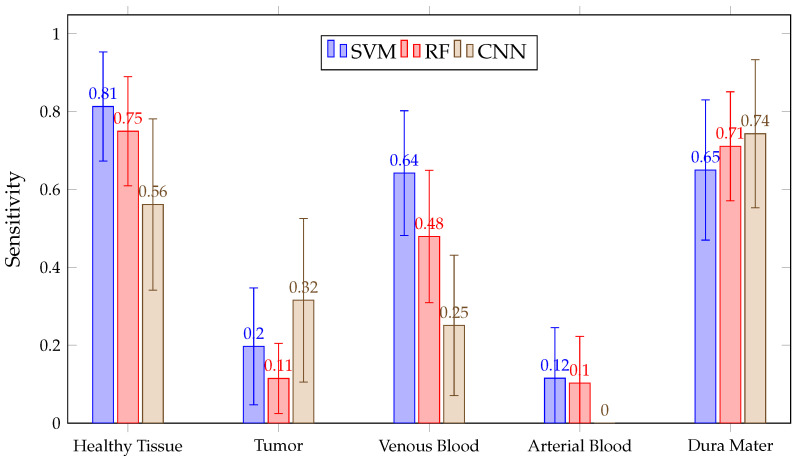
Mean SEN for all patient predictions and every tissue for SVM, RF and CNN algorithms obtained in Experiment B.

Regarding mean SEN values in Figure 18, it can be seen that none of the algorithms correctly differentiate the tumor or arterial blood classes. The authors’ opinion is that this can be explained by looking at Table 1: there are only 1256 pixels for the arterial blood class. Considering that, during double cross-validation, approximately 64% of those pixels (80% for training and 80% of those for calibration) have been used, it might not be using enough arterial blood data to train the models. A similar effect can be observed with the venous blood class. Moreover, for tumor class patients, different tumoral pathologies or tumoral grades have been used and therefore the spectral signatures differ from each other.

**Figure 19:**
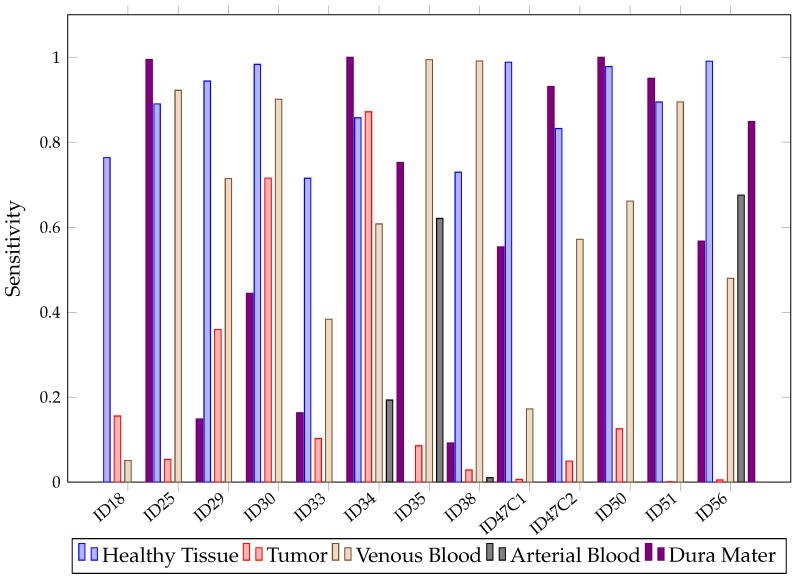
SVM sensibility for each patient and tissue in Experiment B.

Mean SEN values below 32% for tumors are low and not desirable in the operating theater since they indicate that the models are having trouble classifying the class of interest. This situation can be observed in Figure 19, where SEN values for SVM are presented after predicting each patient image. Although SVM achieved healthy tissue SEN values above 70%, most tumor patients were classified below 15%. This might be explained by the high similarities between the mean spectral signatures of the healthy tissue and tumor classes, described in Figure 2 across the 660 to 950 nm measured spectrum. In addition, in Section 4, further measures to increase this ratio are proposed.

With the arterial blood class, it is noted that SVM did not know how to classify it correctly in 10 out of 13 predictions, with 0% values, probably due to the low amount of pixels and high similarity to the venous blood spectral signature. Overall, in Figure 19, it can be observed that, depending on the patient, SVM will classify tumors properly. For patients ID30 and ID34, it classified tumors with 70% and 88% of SEN, but for ID51 and ID56, SEN values were 0%.

Compared SVM with RF and CNN, it is shown that, depending on the patient and the class, algorithms will behave differently, and therefore the best algorithms’ results vary.

**Figure 20:**
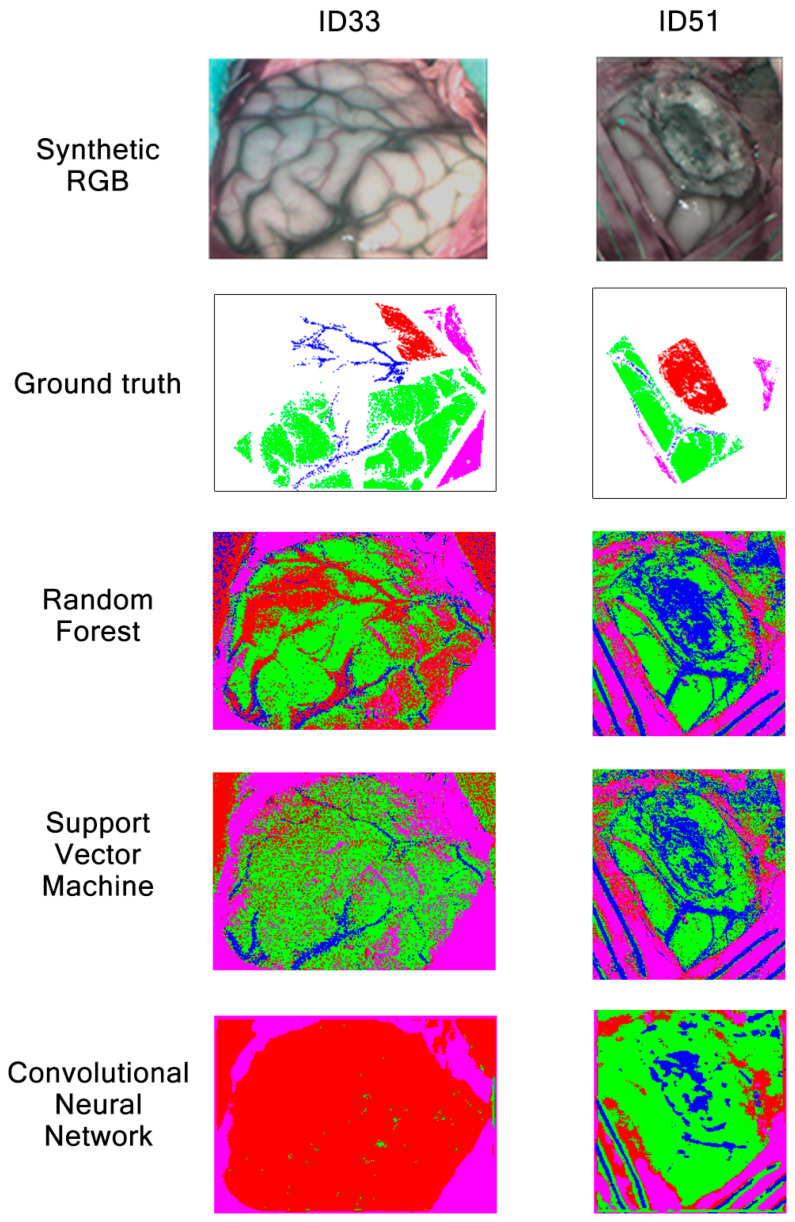
Synthetic RGBs, ground truths and classification maps of patients ID33 and ID51 obtained in Experiment B after being classified by RF, SVM and CNN.

For patients ID33 and ID51, classification maps obtained with RF, SVM and CNN are presented in Figure 20. As already mentioned during the previous classification map analysis in Section 3.1, patients ID33 and ID51 suffer from glioma grade III and astrocytoma grade III tumors, respectively. These results show that all algorithms are good at classifying dura mater, which could be explained due to its mean spectral signature difference among the other classes, as shown in Figure 2. Regarding tumor tissue, all three algorithms have difficulties in classifying it. The CNN classification map for patient ID33 illustrates how this algorithm confuses healthy tissue with tumor, classifying most brain surfaces as tumor. Although RF and SVM also confuse healthy tissue and venous blood as tumor for patient ID33, SVM seems to classify less venous blood pixels as tumor than RF. In any case, these results corroborate the low tumor sensitivity values obtained for the three algorithms in Figure 18. Continuing with image ID51, it is worth noting that this image was captured at an advanced stage in the surgical procedure, where the tumor was located in deeper layers. Results show that any algorithm was able to classify the labeled tumor pixels from the ground truth. This did not happen on the classification maps obtained in Experiment A, where RF and CNN were able to identify tumor in deeper layers. We can see again how the CNN classification map for patient ID51 is less clear than the ones obtained with RF and SVM. Overall, the results show how SVM and RF provided clearer classification maps than CNN for Experiment B.

### 3.3. Comparison with State-of-the-Art Technologies

First, it should be remembered that the proposed methodology consists of a hyperspectral snapshot camera which captures 25 bands included in the red and near IR spectrum. Despite the small number of bands and the similarity between some tissue spectral signatures, as explained in Section 2.3, the OACC results reach 95% using RF in Experiment A and 60% using SVM in Experiment B. These results are presented in Section 3.1 and Section 3.2, respectively.

Regarding brain tumor classification, the state of the art shows that the best results were obtained using a 3D-2D CNN hybrid algorithm, reaching an OACC of 80%, while, using SVM algorithm, it achieved an OACC of 76% [33]. Although, in this work, the best OACC obtained is 60% using the SVM algorithm, OACC drops by 20% in comparison with the 3D-2D CNN hybrid algorithm. However, cameras used in the state of the art present different characteristics to those used in this work. The most notable difference is the number of bands, where, in some articles [33,42], authors used 128 bands; in other works, they used 826 bands [43], instead of the 25 bands that the proposed snapshot camera captures.

In order to compare the accuracy ratio to the number of bands, a metric is proposed (OACC/bands). It indicates the mean OACC per band obtained in each approach used in the state of the art. This metric is presented in Table 4, which shows that the best results are obtained using the proposed methodology in this work, where the OACC ratio per band is 2.4%. Although the 3D-2D CNN hybrid algorithm [33] presents the best results, the proposed metric OACC per band is 0.63%. Therefore, based on this metric, the methodology proposed in this work is 3.81 times better than the best one found in the state-of-the-art glioblastoma classification using HSI.

In addition, some works [33,42] used four classes for classification—normal, tumor, blood vessels and background—while [43] used three classes: normal, tumor and others. In the proposed work, five classes have been classified, so the accuracy results may be affected.

## 4. Conclusions and Future Work

Using HSI techniques in combination with ML algorithms provides a non-invasive and promising method for brain tumor classification, even though the presented results are not extendable to all cases yet. In this work, support vector machine, random forest and convolutional neural networks have been used to classify in-vivo brain tissue in thirteen patients with high-grade glioma pathology.

The mean spectral signature and standard deviation extracted from all 13 images used for the five classes studied show high similarities between healthy and tumor tissue, concretely, across 660 to 950 nm of the measured spectrum. Even though both of them have more than 28,000 and 15,000 samples labeled, respectively, their standard deviation is low compared to the rest of the classes. This might reveal how similar the healthy and tumor tissue are across all 13 images used. In addition, the spectral signatures extracted from venous and arterial blood reveal similarities over the same spectrum region, whereas dura mater seems to be the most differentiated tissue.

From a clinical point of view, the similarity between the signatures of healthy and cancerous tissue could mean that what is being labeled as healthy brain may not be completely healthy tissue. Glioblastomas are highly infiltrative; therefore, the areas surrounding the tumor may present physiological alterations. The lack of divergence between spectral signatures can be problematic for the generalization of the algorithms, mainly because the spectral information is their main source when differentiating one tissue from another. Future work is expected to incorporate a larger number of patients with diverse pathologies, not only oncologic, to enrich the models generated.

Even though these similarities were present in the spectral signatures, Experiment A proved that the three algorithms tested are, indeed, capable of identifying the differences between the five classes. The significant fall in sensitivity in tumor and arterial blood in Experiment B is evidence that alternative ways of training must be explored in future research studies. Incremental learning might help in brain tumor detection. Training an existing model in the operating theater by adding a few pixel samples from the patient undergoing the operation should help during classification. Therefore, it will be considered to implement incremental learning in future works in order to determine whether adding training time while operating on a patient can help in improving brain tumor classification.

On the other hand, in Experiment B, the authors found that SVM achieved 60.1% of OACC on average, whereas RF achieved 52.9% and CNN 49.1%. However, it has been demonstrated that SVM, RF or CNN obtain different results depending on the classified patient and class, potentially due to the inter-patient variability. In addition, it is worth mentioning that none of the algorithms achieved acceptable SEN for tumor tissue, with values below 32%. Thus, improving the process chain with complex and hybrid algorithms to help brain tumor classification is another proposal. Moreover, adding filters as well as more images for the experiments while reducing the dataset size of each image to avoid overfitting are additional research lines which can be considered in the future.

One of the objectives of this work is to advance the development of classification systems that can be used in surgical environments while providing real-time results. Using patients with the same pathologies might also help, since, in this work, we considered high-grade gliomas (GIII and GBM) as the same class. Additionally, further experiments with linescan cameras will be performed to acquire more spectral bands and better spatial resolution to find relevant differences in spectral signatures with the snapshot camera used for this work. Finally, it has been found that having few samples of arterial blood during training might have influenced the algorithms to classify this class. Thus, the authors will consider a minimum set of pixels for every class to ensure good classifications. Other approaches could combine venous blood and arterial blood as a single blood class or even obtain more labeled pixels for the arterial class.

In this work, three classification algorithms have been evaluated using a HSI snapshot camera with a limited number of bands. In addition, the aim was to perform an ambitious classification focused on differentiating between five different tissue types. Taking into account the contribution of each band to the global accuracy, the results obtained are 3.81 times higher than the best results of the state of the art, despite being based on much more robust and complex systems.

## Figures and Tables

**Table 1:** Dataset with the number of total labeled pixels and its percentage.

Patient	Healthy T.	Tumor	Venous B.	Arterial B.	Dura M.	Total
ID18	648 (24.02%)	1587 (58.84%)	79 (2.92%)	14 (0.52%)	369 (13.68%)	2697
ID25	801 (66.42%)	206 (17.08%)	90 (7.46%)	15 (1.24%)	94 (7.79%)	1206
ID29	3752 (67.92%)	64 (1.16%)	98 (1.77%)	11 (0.20%)	1599 (28.95%)	5524
ID30	2587 (34.23%)	2737 (36.21%)	487 (6.44%)	381 (5.04%)	1366 (18.07%)	7558
ID33	6671 (66.95%)	973 (9.76%)	842 (8.45%)	35 (0.35%)	1443 (14.48%)	9964
ID34	1186 (31.32%)	1464 (38.66%)	181 (4.78%)	176 (4.65%)	780 (20.60%)	3787
ID35	3864 (57.38%)	1389 (20.63%)	837 (12.43%)	58 (0.86%)	586 (8.70%)	6734
ID38	1740 (51.85%)	487 (14.51%)	113 (3.37%)	191 (5.69%)	825 (24.58%)	3356
ID47C1	174 (17.17%)	160 (15.79%)	58 (5.73%)	54 (5.33%)	567 (55.97%)	1013
ID47C2	715 (60.19%)	182 (15.32%)	14 (1.18%)	148 (12.46%)	129 (10.86%)	1188
ID50	410 (13.32%)	1282 (41.64%)	712 (23.12%)	47 (1.53%)	628 (20.40%)	3079
ID51	3888 (53.09%)	2731 (37.29%)	95 (1.30%)	85 (1.16%)	525 (7.17%)	7324
ID56	1920 (19.35%)	1967 (19.83%)	75 (0.76%)	37 (0.37%)	5922 (59.69%)	9921

**Table 2:** OACC for SVM, RF and CNN after classifying each patient image in Experiment A.

Patient	Support Vector Machine	Random Forest	Neural Networks
ID18	0.668	0.953	0.916
ID25	0.731	0.968	0.843
ID29	0.947	0.988	0.980
ID30	0.787	0.984	0.956
ID33	0.855	0.960	0.968
ID34	0.831	0.987	0.981
ID35	0.961	0.998	0.996
ID38	0.644	0.949	0.910
ID47C1	0.710	0.927	0.788
ID47C2	0.631	0.885	0.812
ID50	0.562	0.876	0.898
ID51	0.573	0.959	0.961
ID56	0.797	0.964	0.938
MEAN	0.746	0.954	0.919
SD	0.125	0.035	0.062

**Table 3:** OACC for SVM, RF and CNN after classifying each patient image in Experiment B.

Patient	Support Vector Machine	Random Forest	Neural Networks
ID18	0.412	0.287	0.425
ID25	0.680	0.549	0.593
ID29	0.786	0.685	0.835
ID30	0.683	0.635	0.385
ID33	0.666	0.526	0.239
ID34	0.798	0.466	0.615
ID35	0.154	0.113	0.256
ID38	0.552	0.584	0.244
ID47C1	0.701	0.669	0.496
ID47C2	0.623	0.609	0.536
ID50	0.529	0.457	0.474
ID51	0.527	0.560	0.541
ID56	0.705	0.739	0.749
MEAN	0.601	0.529	0.491
SD	0.166	0.158	0.171

**Table 4:** Comparison between OACC results of state-of-the-art approaches.

Approach	Original OACC	N° Bands	OACC/Bands (OACC Per Band)
3D-2D CNN [33]	80%	128	0.63%
3D-2D-CNN + SVM [33]	75%	128	0.59%
SVM [33]	76%	128	0.59%
2D CNN [33]	72%	128	0.56%
1D CNN [33]	78%	128	0.61%
ANN [43]	96.7%	826	0.11%
RF [43]	99.46%	826	0.12%
Spectral Unmixing [42]	76%	128	0.59%
Proposed SVM	60%	25	2.4%
Proposed RF	53%	25	2.12%
Proposed 3DCNN	49%	25	1.96%

## Data Availability

The data presented in this study are available on request from the corresponding author. The data are not publicly available due to privacy reasons.

## Abbreviations

The following abbreviations are used in this manuscript:

GIV	Grade IV glioma
GIII	Grade III glioma
GBM	Glioblastoma multiforme
iMRI	intraoperative Magnetic Resonance Imaging
IOUS	Intraoperative Ultrasounds
5-ALA	5-aminolevunilic acid
MRI	Magnetic Resonance Imaging
HSI	Hyperspectral Imaging
ML	Machine Learning
AI	Artificial Intelligence
DL	Deep Learning
SVM	Support Vector Machine
RF	Random Forest
CNN	Convolutional Neural Network
3DCNN	3D Convolutional Neural Network
DLNN	Deep Learning Neural Network
GT	Ground Truth
SAM	Spectral Angle Mapper
RMS	Root Mean Square
ACC	Accuracy
SEN	Sensitivity
SPE	Specificity
OACC	Overall Accuracy
SD	Standard Deviation
